# A Complete Constellation of Nervous System Lesions of NF2: Imaging Evaluation

**DOI:** 10.1155/2012/353179

**Published:** 2012-03-11

**Authors:** Kiran Gangadhar, Sandeep Kumar, Lovekesh Bhatia, Arjit Agarwal

**Affiliations:** Department of Radiodiagnosis and Imaging, Institute of Medical Sciences, Banaras Hindu University, Varanasi 221005, India

## Abstract

The radiological findings fulfilling the criteria of neurofibromatosis type 2 (NF2) were reviewed. NF2 is a rare disease with few cutaneous but frequent, typical radiological findings in the central nervous system. The presenting symptom is most commonly hearing loss due to acoustic schwannomas, although symptoms emanating from other intracranial or tumors are not uncommon. The discovery of multiple spinal neurofibromas or multiple meningiomas without cutaneous lesions should initiate a search for acoustic schwannomas even when the patient has normal hearing as in our case patient who actually presented for weakness of all four limbs.

## 1. Introduction

Neurofibromatosis (NF) is an inherited autosomal dominant disease mainly affecting the skin, nervous and musculoskeletal system, first described by Von Recklinghausen in 1882. Recently, NF has been divided into two types, defined according to the location of their genetic defect. In NF1, the defect is on chromosome 17, and in NF2, on chromosome 22. NF2 diagnosis might be difficult for the clinician because cutaneous manifestations are often sparse or missing. It is therefore not unusual for the diagnosis to be made by the radiologist. The aim of the present paper is to assess the radiological findings in a patient who fulfilled all the CNS lesions criteria of NF2. 

## 2. Case History

**Figure 1:**
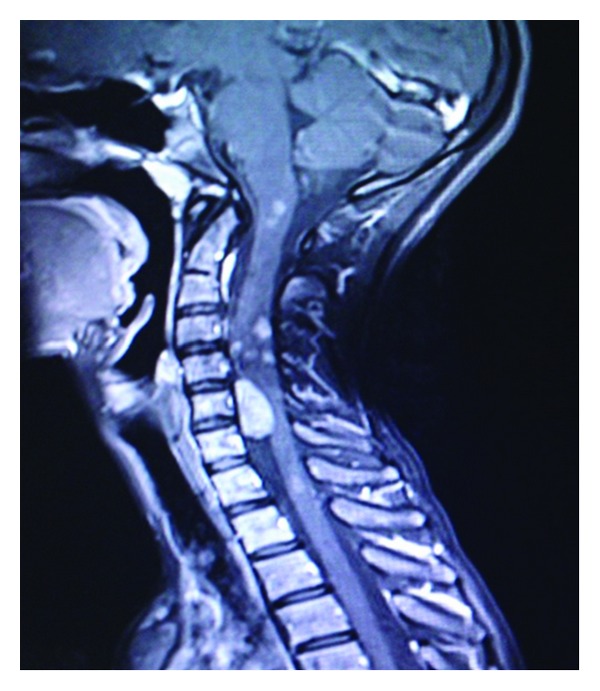
Sagittal T1 postcontrast MR image of the cervical spine showing multiple intramedullary intensely enhancing mass lesions at cervical cord and extramedullary mass lesion at C5 level suggestive of ependymomas/astrocytomas and neurofibroma, respectively.

**Figure 2:**
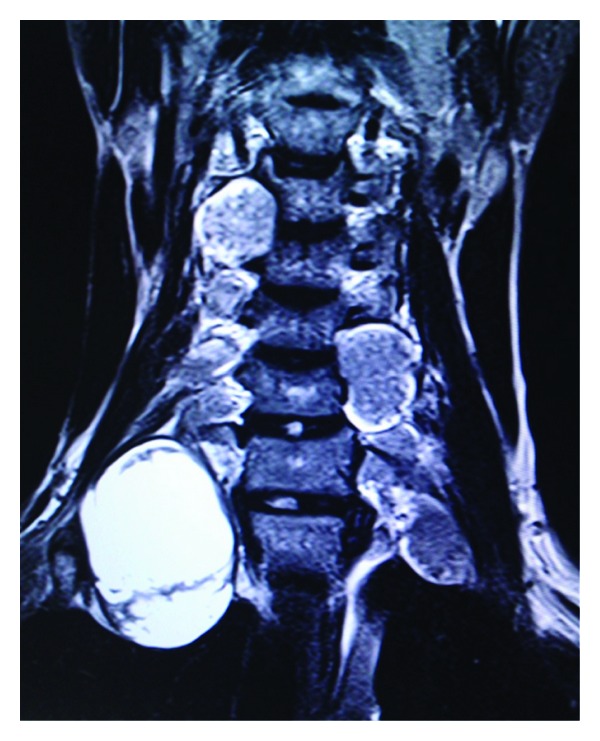
Coronal T2 weighted MR image showing multiple variable sized intra- and extradural dumbbell-shaped tumours in cervical and upper thoracic region suggestive of spinal and extraspinal neurofibromas.

**Figure 3:**
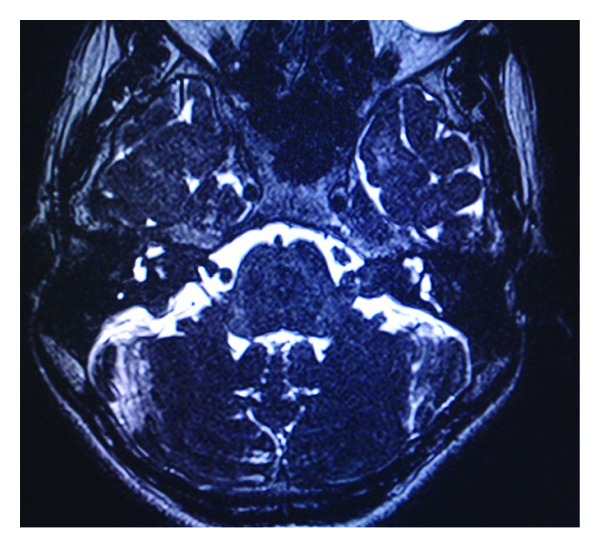
Axial constructive interferance in steady state MR image showing bilateral acoustic schwannomas with internal auditory meatus widening and CP angle cisternal extension.

**Figure 4:**
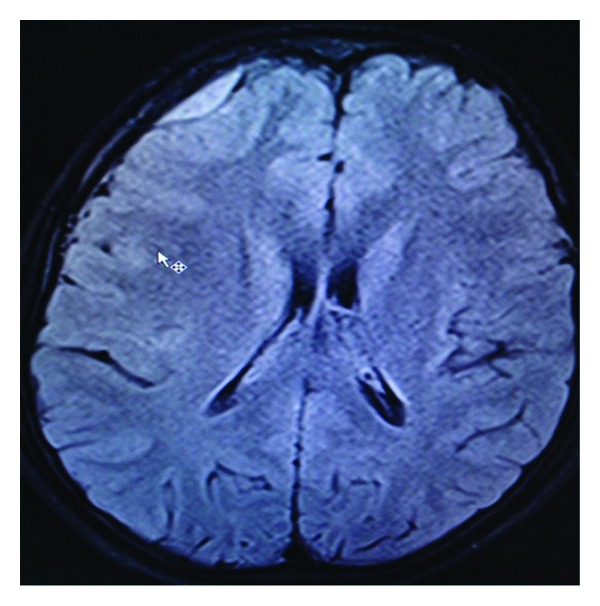
Axial fluid attenuated inversion recovery MR image showing broad dural based extra-axial en plaque meningiomas at right frontal region.

**Figure 5:**
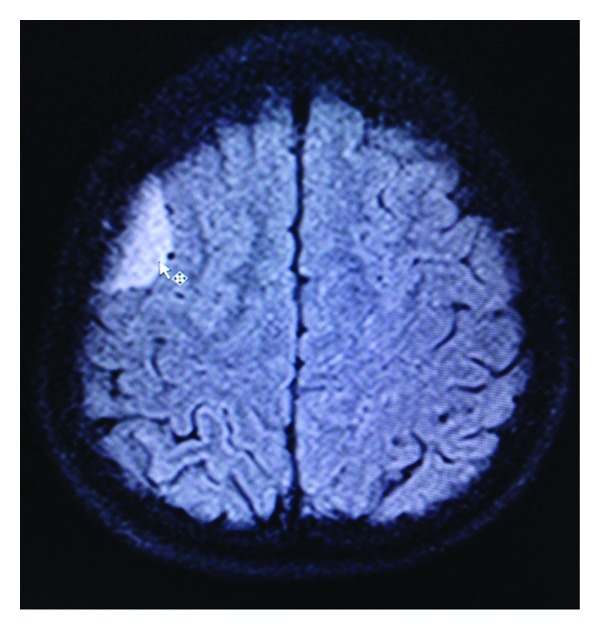
Axial fluid attenuated inversion recovery MR image showing broad dural based extra axial en plaque meningiomas at right frontoparietal region.

**Figure 6:**
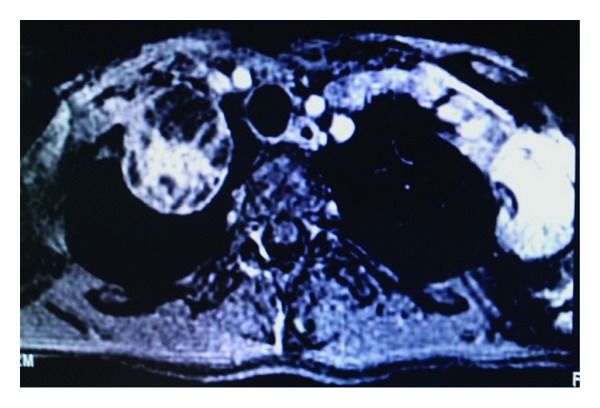
Axial T1 weighted fat saturated postcontrast MR image showing two large extraspinal upper thoracic heterogeneous extraspinal neurofibromas.

An 18-year-old male presented with a history weakness in all four limbs more in upper limbs and chronic headache. Family history was positive for similar complaints and early death of his elder brother. General physical examination revealed an alert and oriented patient, with normal cognitive function, with right lower cervical and left upper thoracic swellings. He had bilateral moderate hearing loss and reduced power in all four limbs. Few scattered skin lesions that resembled skin tags were also found. Imaging evaluation of spine was done, revealed multiple spinal and extraspinal neurofibromas and few intramedullary lesions. Later cranial MR was done, revealed bilateral intracanalicular acoustic schwannomas with two en plaque meningiomas in right frontoparietal region. Considering the CNS lesions and positive family history, a diagnosis of NF2 was done (Figures  1, 2, 3, 4, 5, and 6). 

## 3. Discussion

The term MISME has been proposed to the NF2 syndrome, due to multiple inherited schwannomas (MIS), meningiomas (M), and ependymomas (E). In addition to the neoplasms, posterior subcapsular lenticular opacity (juvenile cortical cataract) is often present. Patients with NF2 may have cutaneous schwannomas that resemble skin tags, but they rarely have caféau lait spots and do not demonstrate the cutaneous neurofibromas like NF1. In 1997, Gutmann et al. proposed revised criteria for diagnostic of the syndrome [1] (Table 1).

The findings of multiple spinal neurofibromas without cutaneous lesions should arise the suspicion of NF2, and examination should be done to rule out acoustic schwannomas even if the hearing is normal. Multiple intracranial meningiomas should initiate a search for acoustic neuromas. In the search for acoustic tumours, MRI with intravenous contrast medium should be used, as its sensitivity is higher than that of other modality [2]. Several authors have studied series of cases to try to define incidence of the tumors in cases of NF2. Mautner et al. studied 48 patients with NF2, in which the prevalence of findings were vestibular schwannomas (CNVIII) in 46 (96%), spinal tumors in 43 (90%), posterior subcapsular cataracts in 30 (63%), meningiomas in 28 (58%), and trigeminal schwannomas in 14 (29%) [3]. Wayman et al. [2] reported cranial MR of 11 patients. In their series, all patients had acoustic schwannomas, 8 had other cranial nerve tumors (5 multiple and 3 single) and 6 had meningiomas (4 multiple and 2 single).

Patronas et al. [4] studied a series of 49 patients with NF2 with spinal MR images, which demonstrated spinal cord and/or canal tumors in 31 (63%). Twenty-six patients (53%) had intramedullary lesions, 27 patients (55%) had intradural extramedullary tumors, and 22 patients (45%) had at least one tumor of each type. Our patient had bilateral CN VIII schwannomas, two enplaque meningiomas, four intracranial and several spinal, and few intramedullary tumors, with no histopathological diagnosis.

## 4. Conclusion

Neurofibromatosis type 2 (NF2) is a rare autosomal dominant disorder with an incidence of 1:37,000 [5]. Its hallmark is the presence of bilateral vestibular schwannomas. Additional manifestations include spinal tumours as well as brain tumours, peripheral nerve tumours, and cataracts [2]. The detection of multiple asymptomatic spinal lesions in patients with a symptomatic spinal tumour led to the assumption that spinal tumours are more common in patients with NF2 than has been assumed [4]. Confirmation of a high frequency of spinal tumours would show that such tumours are criterion of the disease and aid in the diagnosis in family members and sporadic cases with suggested NF2.

## Figures and Tables

**Table 1:** Gutmann et al. [1].

Definite diagnoses of NF2
(i) bilateral CN VIII schwannomas on MRI or CT scan (no biopsy necessary)
(ii) First-degree relative with NF2 and either unilateral early-onset CNVIII schwannoma (age <30 y) or any two of the following:
(1) meningioma
(2) glioma
(3) schwannoma
(4) juvenile posterior subcapsular lenticular opacity (juvenile cortical cataract)

Presumptive diagnoses of NF2
(i) Early onset of unilateral CN VIII schwannomas on MRI or CT scan detected in patients younger than 30 years and one of the following:
(1) meningioma
(2) glioma
(3) schwannoma
(4) juvenile posterior subcapsular lenticular opacity
(ii) Multiple meningiomas (>2) and unilateral CN VIII schwannoma or one of the following:
(1) glioma
(2) schwannoma
(3) juvenile posterior subcapsular lenticular opacity

## Abbreviations

NF2: Neurofibromatosis 2
MR: Magnetic resonance
CNS: Central nervous system
CN: Cranial nerve
CT: Computed tomography.
